# 2-[(Methyl­sulfan­yl)meth­yl]-1,2-benz­isothia­zol-3(2*H*)-one 1,1-dioxide

**DOI:** 10.1107/S1600536808028109

**Published:** 2008-09-06

**Authors:** Waseeq Ahmad Siddiqui, Saeed Ahmad, Hamid Latif Siddiqui, Rana Altaf Hussain, Masood Parvez

**Affiliations:** a Department of Chemistry, University of Sargodha, Sargodha, Pakistan; b Department of Chemistry, University of Science and Technology, Bannu, Pakistan; c Institute of Chemistry, University of the Punjab, Lahore, Pakistan; d Department of Chemistry, University of Calgary, 2500 University Drive NW, Calgary, Alberta, Canada T2N 1N4

## Abstract

In the title mol­ecule, C_9_H_9_NO_3_S_2_, the essentially planar benzisothia­zole ring system and the C—S—C atoms of the methyl­sulfanyl side chain form an angle of 64.45 (7)°. The structure is devoid of any classical hydrogen bonding. However, weak non-classical inter- and intra­molecular hydrogen bonds of the type C—H⋯O are present.

## Related literature

For related literature, see: Bernstein *et al.* (1994 ▶); Masashi *et al.* (1999 ▶); Nagasawa *et al.* (1995 ▶); Siddiqui *et al.* (2007*a* ▶,*b* ▶, 2008*a* ▶,*b* ▶); Xu *et al.* (2006 ▶); Liang (2006 ▶).
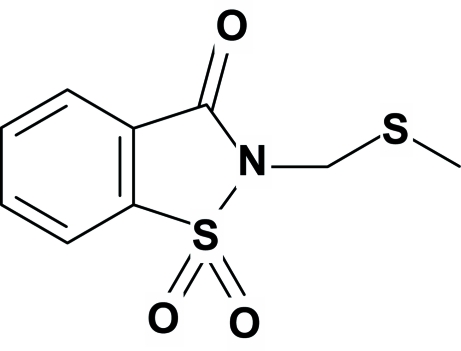


## Experimental

### 

#### Crystal data

C_9_H_9_NO_3_S_2_
*M* *_r_* = 243.29
Monoclinic, 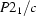

*a* = 7.550 (3) Å
*b* = 17.332 (8) Å
*c* = 9.455 (3) Å
β = 124.26 (2)°
*V* = 1022.6 (7) Å^3^
*Z* = 4
Mo *K*α radiation
μ = 0.51 mm^−1^
*T* = 173 (2) K
0.18 × 0.16 × 0.06 mm

#### Data collection

Nonius KappaCCD diffractometer
Absorption correction: multi-scan (*SORTAV*; Blessing, 1997 ▶) *T*_min_ = 0.915, *T*_max_ = 0.970
3975 measured reflections
2322 independent reflections
2004 reflections with *I* > 2σ(*I*)
*R*_int_ = 0.024

#### Refinement

*R*[*F* ^2^ > 2σ(*F* ^2^)] = 0.035
*wR*(*F* ^2^) = 0.094
*S* = 1.05
2322 reflections
136 parameters
H-atom parameters constrained
Δρ_max_ = 0.29 e Å^−3^
Δρ_min_ = −0.45 e Å^−3^

### 

Data collection: *COLLECT* (Hooft, 1998 ▶); cell refinement: *HKL* *DENZO* (Otwinowski & Minor, 1997 ▶); data reduction: *SCALEPACK* (Otwinowski & Minor, 1997 ▶); program(s) used to solve structure: *SHELXS97* (Sheldrick, 2008 ▶); program(s) used to refine structure: *SHELXL97* (Sheldrick, 2008 ▶); molecular graphics: *ORTEP-3 for Windows* (Farrugia, 1997 ▶); software used to prepare material for publication: *SHELXL97*.

## Supplementary Material

Crystal structure: contains datablocks global, I. DOI: 10.1107/S1600536808028109/lh2683sup1.cif

Structure factors: contains datablocks I. DOI: 10.1107/S1600536808028109/lh2683Isup2.hkl

Additional supplementary materials:  crystallographic information; 3D view; checkCIF report

## Figures and Tables

**Table 1:** Hydrogen-bond geometry (Å, °)

*D*—H⋯*A*	*D*—H	H⋯*A*	*D*⋯*A*	*D*—H⋯*A*
C2—H2⋯O2^i^	0.95	2.49	3.390 (2)	158
C9—H9*B*⋯O3	0.98	2.56	3.383 (3)	142

Symmetry code: (i) 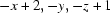.

**Fig. 1.**
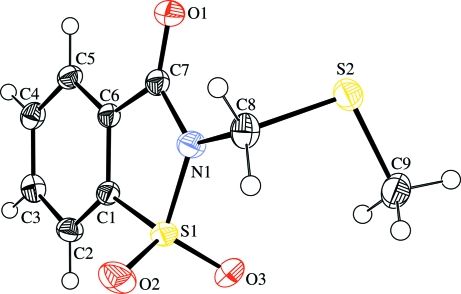
ORTEP-3 (Farrugia, 1997) drawing of the title compound with displacement ellipsoids plotted at 50% probability level.

**Fig. 2.**
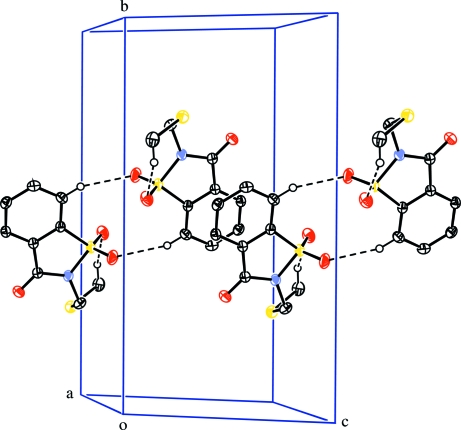
Hydrogen bonding interactions in the unit cell of the title compound indicated by dashed lines, H-atoms not involved in H-bonds have been excluded.

**Fig. 3.**
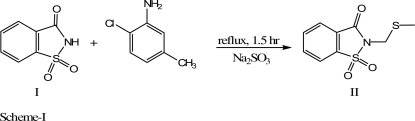
Reaction scheme.

## supplementary crystallographic information

### Comment

1,2-benzisothiazole-3-one 1,1-dioxide (saccharin) has been identified as an important molecular component in various classes of 5-HTla antagonists, analgesics and human mast cell tryptase inhibitors (Liang *et al.*, 2006). Particularly, the substituted derivatives with e.g. *N*-hydroxy and *N*-alkyl substutuents have shown important biological activities (Nagasawa *et al.*, 1995). Various biologically important saccharin skeletons and their *N*-alkyl derivatives were efficiently prepared (Xu *et al.*, 2006) by chromium oxide-catalyzed oxidation of *N*-alkyl(*o*-methyl)arenesulfonamides in acetonitrile besides the already developed methodology utilizing irradiation techniques (Masashi *et al.*, 1999) for similar type of conversions. In continuation of our research program on the synthesis of benzisothiazole derivatives (Siddiqui *et al.*, 2007*a*,*b*,2008*a*,*b*), we report the synthesis (see Fig. 3) and crystal structure of the title compound, in this paper.

In the molecular structure (Fig. 1) the benzisothiazole rings system is essentially planar, the maximum deviation of any atom from the mean plane through S1/N1/C1–C7 being 0.0224 (8) Å for atom S1. The side chain comprising of atoms S2/C8/C9 is inclined at an angle 64.45 (7)° with the mean-plane of the benzisothiazole rings system. The structure is devoid of any classical hydrogen bonding. However, non-classical intermolecular hydrogen bond of the type C—H···O are present resulting in dimeric units in an *R*_2_^2^(8) motif (Bernstein *et al.*, 1994). In addition, intramolecular hydrogen bonds of the type C—H···O are also present in the structure resulting in an *S*(7) pattern (Bernstein *et al.*, 1994) (details are in Fig. 2 and Table 1).

### Experimental

A suspension of saccharin (I) (1.0 g, 5.46 mmol), sodium sulfite (1.4 g, 10.93 mmol) and an excess of 2-chloro-5-methylaniline (5 ml) was first stirred at room temperature (30 min.) and then under reflux (1.5 hrs). The reaction mixture turned orange red after reflux. Cooled the reaction mixture to room temperature and extracted the product with chloroform (3 *X* 25 ml). Concentrated the organic layer under reduced pressure (11 torr) to get light yellow product (II) (0.6 g, 2.46 mmol), yield = 45%. Recrystallization Solvent: MeOH:CH_3_CN (1:1). The solution was subjected to slow evaporation at 313 K to obtain colorless crystals.

### Refinement

Though all the H atoms could be distinguished in the difference Fourier map the H-atoms were included at geometrically idealized positions and refined in riding-model approximation with the following constraints: aryl, methyl and methylene C—H distances were set to 0.95, 0.98 and 0.99 Å, respectively; in all these instances *U*_iso_(H) = 1.2 *U*_eq_(C). The final difference map was free of any chemically significant features.

### Crystal data

**Table:**

C_9_H_9_NO_3_S_2_	*F*(000) = 504
*M**_r_* = 243.29	*D*_x_ = 1.580 Mg m^−^^3^
Monoclinic, *P*2_1_/*c*	Mo *K*α radiation, λ = 0.71073 Å
Hall symbol: -P 2ybc	Cell parameters from 2322 reflections
*a* = 7.550 (3) Å	θ = 4.0–27.5°
*b* = 17.332 (8) Å	µ = 0.51 mm^−^^1^
*c* = 9.455 (3) Å	*T* = 173 K
β = 124.26 (2)°	Plate, colourless
*V* = 1022.6 (7) Å^3^	0.18 × 0.16 × 0.06 mm
*Z* = 4	

### Data collection

**Table:**

Nonius KappaCCD diffractometer	2322 independent reflections
Radiation source: fine-focus sealed tube	2004 reflections with *I* > 2σ(*I*)
graphite	*R*_int_ = 0.024
ω and φ scans	θ_max_ = 27.5°, θ_min_ = 4.0°
Absorption correction: multi-scan (SORTAV; Blessing, 1997)	*h* = −9→9
*T*_min_ = 0.915, *T*_max_ = 0.970	*k* = −22→19
3975 measured reflections	*l* = −12→12

### Refinement

**Table:**

Refinement on *F*^2^	Primary atom site location: structure-invariant direct methods
Least-squares matrix: full	Secondary atom site location: difference Fourier map
*R*[*F*^2^ > 2σ(*F*^2^)] = 0.035	Hydrogen site location: inferred from neighbouring sites
*wR*(*F*^2^) = 0.094	H-atom parameters constrained
*S* = 1.05	*w* = 1/[σ^2^(*F*_o_^2^) + (0.048*P*)^2^ + 0.474*P*] where *P* = (*F*_o_^2^ + 2*F*_c_^2^)/3
2322 reflections	(Δ/σ)_max_ = 0.001
136 parameters	Δρ_max_ = 0.29 e Å^−^^3^
0 restraints	Δρ_min_ = −0.45 e Å^−^^3^

### Special details

**Table:**

Experimental. m.p. 405–406 K; IR (KBr, ν_max_, cm^-1^): CO 1731 (*s*), SO_2_ 1332 and 1177; ^1^H-NMR (400 MHz, DMSO-d_6_) δ: 2.28 (s, 3H, CH_3_), 4.90 (s, 2H, CH_2_), 7.98–8.07 (m, 3H, aromatic), 8.13–8.34 (m, 1H, aromatic); ^13^C-NMR (100 MHz, DMSO-d_6_) δ: 158.3, 136.7, 135.9, 135.3, 125.9, 125.1, 121.6, 42.6, 15.5 LRMS (ES^+^): m/*z*: 244 [*M*]^+^ (63.5%).
Geometry. All e.s.d.'s (except the e.s.d. in the dihedral angle between two l.s. planes) are estimated using the full covariance matrix. The cell e.s.d.'s are taken into account individually in the estimation of e.s.d.'s in distances, angles and torsion angles; correlations between e.s.d.'s in cell parameters are only used when they are defined by crystal symmetry. An approximate (isotropic) treatment of cell e.s.d.'s is used for estimating e.s.d.'s involving l.s. planes.
Refinement. Refinement of *F*^2^ against ALL reflections. The weighted *R*-factor *wR* and goodness of fit *S* are based on *F*^2^, conventional *R*-factors *R* are based on *F*, with *F* set to zero for negative *F*^2^. The threshold expression of *F*^2^ > σ(*F*^2^) is used only for calculating *R*-factors(gt) *etc*. and is not relevant to the choice of reflections for refinement. *R*-factors based on *F*^2^ are statistically about twice as large as those based on *F*, and *R*- factors based on ALL data will be even larger.

### Fractional atomic coordinates and isotropic or equivalent isotropic displacement parameters (Å^2^)

**Table:**

	*x*	*y*	*z*	*U*_iso_*/*U*_eq_	
S1	1.06232 (7)	0.08010 (3)	0.35109 (5)	0.02244 (14)	
S2	1.41986 (7)	0.24291 (3)	0.32581 (6)	0.02667 (14)	
O1	0.9265 (2)	0.19480 (7)	−0.03395 (16)	0.0267 (3)	
O2	0.9634 (2)	0.10390 (9)	0.43595 (17)	0.0340 (3)	
O3	1.2696 (2)	0.04531 (9)	0.45436 (16)	0.0328 (3)	
N1	1.0689 (2)	0.15451 (9)	0.24102 (18)	0.0235 (3)	
C1	0.8872 (3)	0.02719 (10)	0.1645 (2)	0.0197 (3)	
C2	0.8048 (3)	−0.04560 (11)	0.1537 (2)	0.0244 (4)	
H2	0.8436	−0.0741	0.2530	0.029*	
C3	0.6623 (3)	−0.07501 (10)	−0.0102 (2)	0.0255 (4)	
H3	0.6023	−0.1248	−0.0231	0.031*	
C4	0.6060 (3)	−0.03308 (11)	−0.1554 (2)	0.0249 (4)	
H4	0.5069	−0.0543	−0.2655	0.030*	
C5	0.6929 (3)	0.03959 (11)	−0.1413 (2)	0.0224 (4)	
H5	0.6559	0.0680	−0.2404	0.027*	
C6	0.8348 (3)	0.06950 (10)	0.0209 (2)	0.0192 (3)	
C7	0.9421 (3)	0.14577 (10)	0.0633 (2)	0.0202 (3)	
C8	1.1816 (3)	0.22693 (11)	0.3227 (2)	0.0253 (4)	
H8A	1.2215	0.2273	0.4420	0.030*	
H8B	1.0817	0.2704	0.2618	0.030*	
C9	1.6129 (3)	0.18644 (12)	0.5098 (2)	0.0320 (4)	
H9A	1.7529	0.1899	0.5261	0.038*	
H9B	1.5664	0.1325	0.4916	0.038*	
H9C	1.6244	0.2063	0.6117	0.038*	

### Atomic displacement parameters (Å^2^)

**Table:**

	*U*^11^	*U*^22^	*U*^33^	*U*^12^	*U*^13^	*U*^23^
S1	0.0225 (2)	0.0275 (2)	0.0162 (2)	−0.00213 (16)	0.01023 (18)	−0.00010 (16)
S2	0.0260 (3)	0.0289 (3)	0.0266 (2)	−0.00508 (18)	0.0157 (2)	−0.00217 (18)
O1	0.0280 (7)	0.0252 (7)	0.0258 (6)	−0.0001 (5)	0.0145 (6)	0.0052 (5)
O2	0.0433 (8)	0.0417 (8)	0.0282 (7)	−0.0045 (7)	0.0270 (6)	−0.0055 (6)
O3	0.0238 (7)	0.0406 (8)	0.0223 (6)	0.0006 (6)	0.0059 (6)	0.0055 (6)
N1	0.0253 (8)	0.0243 (8)	0.0186 (7)	−0.0056 (6)	0.0109 (6)	−0.0024 (6)
C1	0.0178 (8)	0.0236 (9)	0.0176 (7)	0.0014 (6)	0.0099 (7)	0.0003 (6)
C2	0.0266 (9)	0.0235 (9)	0.0255 (8)	0.0020 (7)	0.0160 (8)	0.0046 (7)
C3	0.0251 (9)	0.0205 (9)	0.0324 (9)	−0.0007 (7)	0.0172 (8)	−0.0016 (7)
C4	0.0222 (8)	0.0275 (9)	0.0212 (8)	−0.0011 (7)	0.0099 (7)	−0.0046 (7)
C5	0.0216 (8)	0.0258 (9)	0.0180 (8)	0.0017 (7)	0.0102 (7)	0.0012 (7)
C6	0.0169 (8)	0.0223 (8)	0.0187 (8)	0.0013 (6)	0.0101 (7)	0.0009 (6)
C7	0.0174 (8)	0.0231 (9)	0.0204 (8)	0.0017 (6)	0.0108 (7)	0.0010 (6)
C8	0.0231 (9)	0.0247 (9)	0.0272 (9)	−0.0027 (7)	0.0135 (8)	−0.0067 (7)
C9	0.0227 (9)	0.0401 (11)	0.0274 (9)	−0.0025 (8)	0.0106 (8)	−0.0025 (8)

### Geometric parameters (Å, °)

**Table:**

S1—O3	1.4304 (15)	C3—C4	1.392 (3)
S1—O2	1.4306 (14)	C3—H3	0.9500
S1—N1	1.6754 (16)	C4—C5	1.392 (3)
S1—C1	1.7537 (18)	C4—H4	0.9500
S2—C8	1.804 (2)	C5—C6	1.385 (2)
S2—C9	1.804 (2)	C5—H5	0.9500
O1—C7	1.208 (2)	C6—C7	1.483 (2)
N1—C7	1.397 (2)	C8—H8A	0.9900
N1—C8	1.468 (2)	C8—H8B	0.9900
C1—C2	1.385 (3)	C9—H9A	0.9800
C1—C6	1.390 (2)	C9—H9B	0.9800
C2—C3	1.393 (3)	C9—H9C	0.9800
C2—H2	0.9500		
			
O3—S1—O2	117.06 (9)	C6—C5—C4	118.30 (16)
O3—S1—N1	110.18 (8)	C6—C5—H5	120.9
O2—S1—N1	109.50 (9)	C4—C5—H5	120.9
O3—S1—C1	112.44 (9)	C5—C6—C1	120.09 (16)
O2—S1—C1	112.28 (9)	C5—C6—C7	126.71 (15)
N1—S1—C1	92.68 (8)	C1—C6—C7	113.20 (15)
C8—S2—C9	100.92 (9)	O1—C7—N1	123.12 (16)
C7—N1—C8	121.81 (15)	O1—C7—C6	128.06 (15)
C7—N1—S1	115.07 (12)	N1—C7—C6	108.81 (14)
C8—N1—S1	122.85 (12)	N1—C8—S2	114.65 (12)
C2—C1—C6	122.64 (16)	N1—C8—H8A	108.6
C2—C1—S1	127.13 (13)	S2—C8—H8A	108.6
C6—C1—S1	110.22 (13)	N1—C8—H8B	108.6
C1—C2—C3	116.69 (16)	S2—C8—H8B	108.6
C1—C2—H2	121.7	H8A—C8—H8B	107.6
C3—C2—H2	121.7	S2—C9—H9A	109.5
C2—C3—C4	121.45 (17)	S2—C9—H9B	109.5
C2—C3—H3	119.3	H9A—C9—H9B	109.5
C4—C3—H3	119.3	S2—C9—H9C	109.5
C5—C4—C3	120.82 (16)	H9A—C9—H9C	109.5
C5—C4—H4	119.6	H9B—C9—H9C	109.5
C3—C4—H4	119.6		
			
O3—S1—N1—C7	116.17 (13)	C4—C5—C6—C1	0.0 (3)
O2—S1—N1—C7	−113.73 (13)	C4—C5—C6—C7	−179.11 (16)
C1—S1—N1—C7	1.06 (13)	C2—C1—C6—C5	0.8 (3)
O3—S1—N1—C8	−69.74 (16)	S1—C1—C6—C5	−178.13 (13)
O2—S1—N1—C8	60.36 (16)	C2—C1—C6—C7	−179.96 (15)
C1—S1—N1—C8	175.15 (14)	S1—C1—C6—C7	1.10 (18)
O3—S1—C1—C2	66.76 (18)	C8—N1—C7—O1	4.5 (3)
O2—S1—C1—C2	−67.75 (18)	S1—N1—C7—O1	178.63 (14)
N1—S1—C1—C2	179.89 (16)	C8—N1—C7—C6	−174.75 (14)
O3—S1—C1—C6	−114.36 (13)	S1—N1—C7—C6	−0.59 (17)
O2—S1—C1—C6	111.13 (13)	C5—C6—C7—O1	−0.4 (3)
N1—S1—C1—C6	−1.22 (13)	C1—C6—C7—O1	−179.53 (17)
C6—C1—C2—C3	−0.8 (3)	C5—C6—C7—N1	178.80 (16)
S1—C1—C2—C3	177.98 (13)	C1—C6—C7—N1	−0.37 (19)
C1—C2—C3—C4	0.0 (3)	C7—N1—C8—S2	−76.41 (19)
C2—C3—C4—C5	0.8 (3)	S1—N1—C8—S2	109.89 (14)
C3—C4—C5—C6	−0.8 (3)	C9—S2—C8—N1	−82.19 (15)

### Hydrogen-bond geometry (Å, °)

**Table:**

*D*—H···*A*	*D*—H	H···*A*	*D*···*A*	*D*—H···*A*
C2—H2···O2^i^	0.95	2.49	3.390 (2)	158
C9—H9B···O3	0.98	2.56	3.383 (3)	142

Symmetry codes: (i) −*x*+2, −*y*, −*z*+1.

## References

[bb1] Bernstein, J., Etter, M. C. & Leiserowitz, L. (1994). *Structure Correlation*, Vol. 2, edited by H.-B. Bürgi & J. D. Dunitz, pp. 431–507. New York: VCH.

[bb2] Blessing, R. H. (1997). *J. Appl. Cryst.* **30**, 421–426.

[bb3] Farrugia, L. J. (1997). *J. Appl. Cryst.* **30**, 565.

[bb4] Hooft, R. (1998). *COLLECT* Nonius BV, Delft, The Netherlands.

[bb5] Liang, X., Hong, S., Ying, L., Suhong, Z. & Mark, L. T. (2006). *Tetrahedron*, **62**, 7902–7910

[bb6] Masashi, K., Hideo, T., Kentaro, Y. & Masataka, Y. (1999). *Tetrahedron*, **55**, 14885–14900.

[bb7] Nagasawa, H. T., Kawle, S. P., Elberling, J. A., DeMaster, E. G. & Fukuto, J. M. (1995). *J. Med. Chem.* **38**, 1865–1871. 10.1021/jm00011a005 7783118

[bb8] Otwinowski, Z. & Minor, W. (1997). *Methods in Enzymology*, Vol. 276, *Macromolecular Crystallography*, Part A, edited by C. W. Carter Jr & R. M. Sweet, pp. 307–326. New York: Academic Press.

[bb9] Sheldrick, G. M. (2008). *Acta Cryst.* A**64**, 112–122. 10.1107/S0108767307043930 18156677

[bb10] Siddiqui, W. A., Ahmad, S., Khan, I. U., Siddiqui, H. L. & Parvez, M. (2007*a*). *Acta Cryst.* E**63**, o4116.

[bb11] Siddiqui, W. A., Ahmad, S., Siddiqui, H. L. & Parvez, M. (2008*a*). *Acta Cryst.* E**64**, o724. 10.1107/S1600536808004637 PMC2960984 21202114

[bb12] Siddiqui, W. A., Ahmad, S., Siddiqui, H. L., Parvez, M. & Rashid, R. (2008*b*). *Acta Cryst.* E**64**, o859. 10.1107/S1600536808009951 PMC2961185 21202346

[bb13] Siddiqui, W. A., Ahmad, S., Siddiqui, H. L., Tariq, M. I. & Parvez, M. (2007*b*). *Acta Cryst.* E**63**, o4001.

[bb14] Xu, L., Shu, H., Liu, Y., Zhang, S. & Trudell, M. (2006). *Tetrahedron*, **62**, 7902–7910.

